# Medical Management for Fracture Prevention in Children with Osteogenesis Imperfecta

**DOI:** 10.1007/s00223-024-01202-7

**Published:** 2024-03-29

**Authors:** Paul Arundel, Nick Bishop

**Affiliations:** 1 Sheffield Children’s NHS FT, Sheffield, UK; 2 Division of Clinical Medicine, School of Medicine and Population Health, University of Sheffield, Sheffield, UK

**Keywords:** Bisphosphonate, Denosumab, Anti-sclerostin antibody, Anti-TGFb antibody, Mesenchymal stem cell therapy

## Abstract

There are no licensed treatments for children with osteogenesis imperfecta. Children currently receive off-label treatment with bisphosphonates, without any consistent approach to dose, drug or route of administration. Meta-analyses suggest that anti-fracture efficacy of such interventions is equivocal. New therapies are undergoing clinical trials, and it is likely that one or more will receive marketing authorisation within the next three to five years. The long-term outcome from such interventions will need to be studied carefully well beyond the period over which the clinical trials are conducted, and a consistent approach to the collection of data in this regard will be needed as a major collaborative effort.

## Background

Most fractures in children happen when they fall, run into something, or something runs into them. The variation in fracture frequency with age and growth rate, and the difference in overall rates between the sexes are well documented. To summarise, boys fracture more than girls at all ages from two years onwards, fracture rate rises throughout childhood, peaking at around the time of maximum height velocity and then declining [1]. Most children do not fracture; around 20% of children will fracture more than once [2]. Fracture risk is associated with bone mass and size—children with larger and more massive bones relative to their body size have lower risk [3]—but irrespective of either bone size or mass, risk increases with the amount of vigorous activity undertaken by the child [4].

When considering fracture risk in the child with osteogenesis imperfecta (OI), some other factors should be considered; fracture risk is intrinsically higher due to multiple factors: the abnormal material property of the bone tissue—the increased mineralisation density along with increased intermolecular cross-linkage means that dissipation of force within bone is much reduced, i.e. it is brittle [5]; there is less trabecular bone, cortices are thinner and there is increased cortical porosity [6]; there is increased bone turnover; bones are narrower; and bones may be deformed.[7].

In trying to reduce fracture risk, the treating team will consider these factors, providing lifestyle advice as well as dispensing pharmacological interventions. Whilst fractures remain a key outcome on which regulatory authorities focus when determining treatment efficacy, there are other outcomes that matter to patients and their families [8]. These include overall quality of life, mobility, pain, fatigue, school attendance, the development of skeletal deformity in particular scoliosis and basilar invagination, as well as the risk of adverse effects of treatment. Of course, some of these outcomes are themselves directly impacted by fractures and their complications.

## Pharmacological Interventions in Children with OI

A range of pharmacological interventions to reduce fracture risk in OI have been investigated over the course of several decades. In the clinical space, this has always been in the context of optimisation of multidisciplinary care alongside medical management. We review these interventions according to their modality of action.

## Antiresorptive Pharmacological Interventions in Children with OI

### Calcitonin

In 1979, a prospective open-label study of salmon calcitonin was published. Calcitonin 2 units was administered three times per week subcutaneously for 6–48 months, along with a daily calcium supplement of 230–345 mg in 48 children and 2 young adults clinically diagnosed with OI [9]. The authors reported increased phalangeal bone density (using photodensitometry and a calibrating aluminium wedge) in those less than age five years compared to healthy controls; older children showed lower gain in bone density compared to controls. The authors reported reduced fracture frequency during the period of the study compared with the fracture rate in the three years preceding treatment across all age groups and severities of OI, as well as improved (self-reported) motor performance in the majority. Five patients withdrew due to lack of tolerance or adverse reactions. There was no evidence of any rebound phenomenon.

### Bisphosphonates

Over the last 30 or so years, the mainstay of pharmacological treatment has been the use of bisphosphonates; however, there has been little consistency between centres in the choice of drug (including route of administration), starting or stopping criteria, monitoring (other than 6 or 12 monthly DXA scans), or dose and dose adjustment criteria. Such variability and lack of consistency can be attributed to some degree to the difficulties in undertaking placebo-controlled trials of intravenous medicines in children, and to the prevailing climate in drug regulation at the time when pamidronate use in children with OI began to be reported. There were no incentives at that time for pharma companies to undertake studies in children, the paediatric market was small and the risk of unanticipated side effects that might impact on sales in the adult market was unknown. All these factors combined meant that there were no pharma-led studies of intravenous bisphosphonates, and the only marketing authorisation applied for was for neridronate in Italy. Thus, no licence was given with labelling that specified when, how much, and for how long treatment with the more commonly used bisphosphonates such as pamidronate and zoledronic acid should be considered and undertaken. Although a pharma-led study of zoledronic acid vs pamidronate in children with OI (NCT00063479) was undertaken from 2003 onwards, no peer-reviewed publication has emerged and no results are listed on the Clinicaltrials.gov website.

Investigator-led studies of bisphosphonate use were initially therefore of open-label studies in relatively small numbers of children reporting primarily on changes in areal bone mineral density, typically of the lumbar spine (LSaBMD). The first report of any bisphosphonate use in OI was in 1987 [10], with oral pamidronate 250 mg/d being given for two months “on” and two months “off” to a 12-year-old girl for a year. The characteristic para-metaphyseal lines were observed, and there was clear increase in the height of previously crush-fractured vertebrae. Another case report followed in 1988, again of treatment with oral pamidronate, this time of 100 mg daily in a severely affected 12-year-old boy weighing only 10 kg [11]. Again increased metaphyseal density was observed and substantially reduced (compared to pre-treatment) fracture frequency was reported—one fracture during the six-month period of treatment, as opposed to two or more fractures per month in the preceding year.

These encouraging reports led a number of centres to start using pamidronate either orally or intravenously; observational cohort studies reported on beneficial effects in terms of increased bone mineral density, cortical thickness, vertebral shape, and mobility [12–16].

At a tissue level, detailed studies of bone biopsies comparing the effects of bisphosphonate treatment within patients, i.e. at baseline and after 2 or more years, and against age-matched controls were reported, primarily from Montreal, Canada. Bone histomorphometric analysis showed slowing of bone turnover, increased bone volume in both the trabecular and cortical compartments and increased cortical thickness due to reduced endocortical resorption [17, 18]. Thus, bisphosphonate treatment in children with OI was shown to favourably alter a number of factors known to be associated with fracture risk reduction.

No controlled trials were reported, possibly for the reasons stated previously, until the mid-2000s when studies of the use of oral and intravenous bisphosphonates in placebo or comparator trials were published. In the only placebo-controlled trial of intravenous therapy (neridronate) in children, the total number of fractures in the untreated group was greater during the placebo-controlled period, but not the number of patients experiencing a fracture [19].

A single dose-ranging study of intravenous pamidronate in infants showed greater LSaBMD increase in those receiving 12 mg as opposed to 6 mg over the one year study period; notably vertebral crush fractures improved or remained unchanged in all infants [20].

A 2012 comparator study of pamidronate and zoledronic acid reported similar increases in LSaBMD after one year using “typical” doses of each drug and no difference in fracture frequency [21]; a pharma-run study undertaking a similar comparison in children aged 3 months to 17 years was stopped in 2007 by its Data Monitoring and Ethics Committee after fully recruiting due to an excess of femoral fractures in the zoledronic acid group and has never reported.

Table 1 summarises the placebo-controlled and comparator studies undertaken using intravenously administered bisphosphonates.

**Table 1:** Controlled trials of intravenous bisphosphonates, including comparator studies with oral bisphosphonates and “add on” treatments, in children

References	Number of each OI type, total no. subjects	Study design	Drug, dose	Duration	Outcomes reported	LSaBMD % or z-score change over initial 12 months	Comment
Letocha et al. [81]	9 Type III 9 Type IV *n* = 18	Controlled RCT. No stratification or minimisation applied. Control group not infused with placebo	Pamidronate 10 mg/m^2^/d 3 monthly ± growth hormone 0.06 mg/kg/d for 6 days/week (*n* = 4 in each group)	1 year	LSaBMD, spine QCT, vertebral area from lateral spine radiographs, fractures	+ 1.43 SDS versus controls	No difference in growth rates between groups. No change in bone formation markers; bone resorption markers not reported. Increased LSaBMD and vertebral size, reduced upper limb fracture frequency in pamidronate-treated group (22 versus 44% in placebo group). No change in functional outcomes of pain or motor function
Gatti et al. [19]	42 Type I; 4 Type III; 18 Type IV *n* = 64	Prepubertal age 6–11 years Open-label RCT 2:1 active: no treatment Control group not infused with placebo	Neridronate, 8 mg/kg/yr given as 2 mg/kg 3 monthly versus no treatment	1 year, 2 years open-label extension	LSaBMD and Hip aBMD Spine anteroposterior and lateral radiographs for vertebral deformity Bone formation—BSALP Fractures	Approximately 24–26% increase versus controls	Results to 12 months in treated versus untreated group: increased spine bone area 6.68% versus 1.97%; increased LSaBMD (30% versus 5%, approximately); reduced BSALP (20%) Fracture relative risk after 12 months, 0.36; 95% CI, 0.15–0.87; *p* < 0.05
DiMeglio and Peacock [82]	12 Type I; 6 type III/IV *n* = 18	Oral vs iv Open-label RCT stratified by age and pubertal status and OI severity	Alendronate 10–20 mg/d oral versus Pamidronate 9 mg/kg/yr [4 monthly infusions over 3 days]	2 years	LSaBMD, fractures, bone turnover markers, growth	+ 1.4 SDS in oral group; + 1.1 SDS in intravenous group	LSaBMD and TBaBMD z-score increased in both groups progressively; similar reductions in NTX, ALP between groups versus baseline NTX 54% in iv and 61% in oral group; BSALP 26% in iv and 33% in oral group. No difference in fracture frequency, which was reduced during the study period across entire cohort compared to one year prior
Senthilnathan et al [20]	Type III/IV not differentiated infants *n* = 12	Dose-ranging blinded RCT No placebo group	Pamidronate 6 versus 12 mg/kg/yr given in incrementally increasing doses over 2 days	1 year	LSaBMD, vertebral size, crush fractures, skeletal survey, bone turnover markers	108% increase from baseline in 6 mg/kg group; 153% increase in 12 mg/kg group; note—expected 50–60% increase in LSaBMD over first year of life in normal infants	Increased bone mass in higher dose group, vertebral height increased in 11/12, increased more in 12 mg group. NTX fell by 74% and ALP 28% by over the 12 months. All infants increased their length z-score from baseline
Barros et al [21]	3 Type I; 14 Type III; 6 Type IV *N* = 23	Open label, randomised to drug, age stratification	Pamidronate 6 (> 3 yrs) or 8 (< 3 yrs) mg/kg/yr vs Zoledronate 0.2 (< 3 yrs) or 0.3 (> 3 yrs) mg/kg/yr	1 year	LSaBMD, TBaBMD, bone biochemistry and bone turnover markers	52% increase over baseline in pamidronate group; 75% increase over baseline in zoledronic acid group	Increased LSaBMD in both groups versus baseline; no difference between treatment groups. Stated that fracture rate had reduced in both groups versus pre-treatment rate, but data not shown

Dose-ranging and placebo-controlled trials of oral medication were reported from 2004 onwards and are summarised in Table 2 [22–27].

**Table 2:** Controlled trials of oral bisphosphonates in children

References	Number of each OI type, total no. subjects	Study design	Drug, dose	Duration	Outcomes reported	LSaBMD % or z-score change over initial 12 months	Comment
Sakkers et al [22]	13 type I; 9 type III; 12 type IV. 11 non-walkers *n* = 34	Placebo-controlled RCT No stratification	Olpadronate 10 mg/m^2^	2 years	LSaBMD, radiologically confirmed incident non-vertebral fractures, bone turnover markers, growth, functional measures—muscle strength, paediatric evaluation of disability inventory (PEDI), Bleck score	Unadjusted 15% greater change in treated group; adjusted for age, height, weight, OI type, sex and pubertal stage 17%	Results to 24 months in treated versus placebo group, both unadjusted and adjusted for age, body size, pubertal status, sex and OI type: significant increase in spine BMC and aBMD; no difference in vertebral heights, growth, muscle strength at hand, shoulder or hip, functional score (PEDI score), Bleck score or bone turnover markers. Reduction in radiologically confirmed incident non-vertebral fracture frequency; hazard ratio 0.69 (95% CI 0.52—0.91; *p* = 0.01)
Seikaly et al [23]	2 Type I; 8 Type III; 10 Type IV *n* = 20	Crossover double blind RCT versus placebo	Alendronate 5 or 10 mg/d (< or ≥ 30 kg)—alendronate pulverised and encapsulated	12 months each arm	Quality of life (PEDI mobility domain, WeeFIM, pain score), bone turnover markers, LSaBMD, height	1.01 SDS increase in treated compared to placebo group	Results to 12 months in treated vs placebo group Significantly improved height, LSaBMD z-score, and all QoL measures except mobility score. No change in bone formation markers; reduced urinary NTX. No clear effect on fracture incidence
Rauch et al [24]	26 Type I *n* = 26	Placebo-controlled double blind RCT 1:1 active: placebo Stratified by weight	Risedronate 15 mg (< 40 kg) or 30 mg (> 40 kg)/week	2 years	LSaBMD, TBaBMD, Hip aBMD, radial pQCT, fractures, bone turnover markers, growth	23.8% versus 6.8% treated versus control	Results to 24 months in treated versus placebo group: increased LSaBMD z-score 0.65 versus − 0.15; no differences in radial pQCT measures; NTX reduced by 35% versus 6%; no difference in grip strength, vertebral morphometry or fracture rate
Bishop et al [26]	1 Type 1; 7 Type III; 45 Type IV; *n* = 53	Dose-ranging open-label RCT 1:1:1	Risedronate; 0.2 mg versus 1 mg versus 2 mg/kg/week	2 years	LSaBMD, TBaBMD, bone turnover markers, growth, vertebral size and shape, scoliosis, fractures	16.5% difference between 2 and 0.2 mg/kg groups after 2 years	Results to 24 months in treated versus placebo group: 0.2 mg/kg group reduced, 1 mg/kg group maintained and 2 mg/kg/d group increased size-adjusted LSaBMD. No difference between groups in fracture frequency, vertebral size or shape, growth or scoliosis
Ward et al [27]	26 Type 1; 32 Type III; 37 Type IV; 14 Unknown type *n* = 139	Placebo-controlled double blind RCT 3:1 active: placebo	Alendronate 5 or 10 mg/d stratified by weight < / ≥ 40 kg	2 years	LSaBMD, TBaBMD bone turnover markers, vertebral and long bone fractures, growth	50.7% vs 11.9% increase over 24 months; 1.32 versus 0.14 SDS	Results to 24 months in treated versus placebo group: Increased LSaBMD z-score (+ 1.32 versus + 0.14 *p* < 0.001; adjusted for baseline, centre and stratum); no difference in fractures; reduced NTX
Bishop et al [25]	12 1Type 1; 5 Type III; ` 17 Type IV; *n* = 147	Placebo-controlled double blind RCT 2:1 active: placebo	Risedronate 2.5 or 5 mg/d stratified by weight ≤ / > 30 kg	1 year blinded, 2 years open label	LSaBMD, TBaBMD, bone turnover markers, growth, fractures	0.427 versus − 0.008 SDS	Results to 12 months in treated versus placebo group: Increased LSaBMD z-score (+ 0.43 versus 0; *p* < 0.0001) and TBaBMD (+ 0.26 versus 0; *p* = 0.03), lower turnover, reduced non-vertebral fracture frequency (31 versus 49% treated versus placebo; *p* = 0.04); reduced NTX and ALP

Meta-analyses of bisphosphonate treatment in OI have suggested that anti-fracture efficacy is equivocal [28, 29]; however, the range of interventions in terms of drug, dose and duration of therapy is large and the severity of the underlying disease and age of the study participants have been additional factors likely to have influenced outcomes. It is worth noting that in the two studies reporting a reduction in fracture frequency with the use of oral bisphosphonates [22, 25], the underlying disease severity was “mild” in the majority of children studied, i.e. most had type I OI; a third smaller study of children with type I OI showed no difference in fracture risk [24]. In the two studies reporting oral therapy in more severely affected children—one dose-ranging [26], the other placebo-controlled [27]—fractures were reported but no risk reduction was shown. However, LSaBMD increased progressively with dose of bisphosphonate administered in the dose-ranging study and increased also in treated patients in the placebo-controlled trial.

Despite the lack of clear anti-fracture efficacy, bisphosphonates are currently the most widely used intervention in children with OI. Bisphosphonates are typically started: when long bone fractures occur often enough to interfere with mobility and school attendance; when vertebrae are observed to be deformed consistent with crush-fracturing; when there is intractable bone pain; or in infants with fractures that have occurred within the first six months of life, including in utero and perinatal fractures. Some centres will also start treatment in infants with OI Type V before any fractures occur given the frequency of vertebral fractures in the first year of life.

The drugs most frequently used in Europe, the Americas, and the Asia–Pacific region are pamidronate and zoledronic acid intravenously, and alendronate and risedronate orally. The total annual doses of pamidronate vary from 3 to 12 mg/kg/year; for zoledronic acid the total annual dose is usually 0.1 mg/kg/year. Alendronate 2 mg/kg and risedronate 1 mg/kg are usually administered at weekly intervals; risedronate 2 mg/kg/week has been reported to be safe and associated with greater gain in LSaBMD compared to 1 mg/kg/week [26].

Treatment is usually commenced using the intravenous formulations; some centres continue to use pamidronate in infants and young children initially, moving to zoledronic acid as they grow, and others use zoledronic acid from the beginning. [30, 31] The main advantage of zoledronic acid is ease of administration—infusions last one hour with another few minutes to flush through; pamidronate is typically given over 2–3 successive days, each infusion lasting four hours plus time for the flush of the giving set. Depending on the severity of the child’s condition and the configuration of services, the shorter infusions of zoledronic acid may result in either more or less time being freed up for providing therapy and nursing inputs, and to undertake investigations e.g. DXA scans. It may seem counter intuitive that a shorter infusion may allow less time for other aspects of care. However, if the intervention takes place on a single day only (rather than over 2–3 days), then the timing and coordination of therapy sessions, specialist nursing activity, DXA scan and administration of drug—particularly if the child’s weight on the day is required before the infusion is made up in pharmacy—can make the schedule of an admission very crowded. In addition, travel time for families constitutes a greater proportion of the time commitment for shorter admissions. This and other factors may come more into play if expectations are not managed carefully e.g. parents may think that they can maintain late afternoon commitments such as school pick-ups and therefore be reluctant to stay long enough to obtain the full value of an admission. For most major centres, more than one child is being treated each day, so organisation of care, whilst complex, becomes crucial to ensuring that children with OI gain the full benefit of medical treatment.

Modern DXA scanners usually provide the treating team with data for both the lumbar spine and total body—hip scans are less reliable in children due to the evolving anatomy of the region and the difficulties of repositioning. In addition, lateral spine images can be obtained, allowing the assessment of changes in the shape of crush-fractured vertebrae with lower doses of radiation than conventional plain radiography [32, 33]. Lateral vertebral scans using DXA are particularly useful for assessing the upper thoracic vertebrae, which are often less well visualised on plain radiographs. Scoliosis can make assessment of crush fractures difficult irrespective of the imaging modality.

Some centres digitally remove lumbar vertebrae that are crush-fractured from the region of interest, and some also digitally mask intramedullary rods before assessing whole body images using software supplied with the DXA machine. Some centres routinely select analyses which exclude vertebrae more commonly subject to fracture e.g. preferring to use DXA L2-4 bone mineral density (BMD) rather than L1-4 BMD data. It is important that for an individual child, a consistent approach is adopted. Sudden changes in bone density results are usually artefactual—typically due to the region of interest “mask” being incorrectly applied, positioning issues, or an alteration in analysis methodology such as a software “upgrade”.

Dose of treatment being given may be adjusted based on either the absolute DXA-derived LSaBMD Z-score, or the trajectory of the Z-score. In general, treatment dose is almost always reduced when LSaBMD Z-score exceeds + 2.0, but some centres reduce the dose well before this point is reached (based on personal communications). Unusually high values may be an indicator of “high bone mass OI” [34]. Overall treatment dose may be decreased by a reduction in the dose infused, an increase in the interval between infusions, or a combination of both [35]. There is no consensus on whether any particular method of reduction is safer or more effective.

Stopping treatment is not usually considered until final height or near final height is achieved [36], unless there is evidence of intolerance or severe adverse reaction. When final height is reached, some centres will stop treatment and review the patient after six months; some will offer a “weaning off” regimen, and some will offer a switch from intravenous to oral treatment. At an anecdotal level, the experience in Sheffield is that some of those in whom treatment is stopped (after cessation of growth) will ask for it to be restarted after six months, the usual reason being “lack of energy”, musculoskeletal pain and/or fatigue. Most individuals who restart treatment using an oral bisphosphonate in this situation seem to feel that it meets their needs in terms of symptomatic improvement; a small minority request the reinstatement of their intravenous therapy. A meta-analysis of the use of bisphosphonates for pain relief in children with bone problems concluded that they have some efficacy, but that the available studies were at high risk of bias [37]. Stopping treatment before growth ceases may result in the new bone formed being of reduced mass and density compared to bone accrued during the treatment period, with the consequent risk of fractures at the interface between the “old” and “new” bone [38].

The practice of monitoring of bisphosphonate treatment in children with OI using bone turnover markers is very variable. In part, this may reflect practical difficulties in relation to sample collection and the consequent “noisiness” of data seen in real-world practice, but may also reflect the uncertainty regarding the utility of measurements even when samples are collected correctly and consistently. A lack of normative data in children for the latest bone turnover markers adds to the difficulty in interpretation of measurements in individual cases. Whilst monitoring bone turnover markers in individuals over time may have some value in terms of monitoring for “over-suppression” of bone remodelling with bisphosphonate treatment, in fact, such “over-suppression”, reflected in abnormally low bone turnover markers, has not been reported [25].

Some centres prefer to regard the complete reshaping of vertebral bodies as a more biologically meaningful outcome to judge when treatment adjustment should be considered [30]. Administration of bisphosphonates from an early age is associated with a delay in the appearance of scoliosis [39] and also reduced risk of scoliosis in patients with mild disease [40]; however, it is unclear whether bisphosphonate-associated vertebral reshaping reduces scoliosis risk overall.

Growth is not impaired by bisphosphonate use; there are some suggestions that preserving or restoring vertebral height may improve height overall in moderate OI [41].

Multidisciplinary input has undoubtedly had an effect on the overall outcomes for children with OI [42], and it is difficult in observational studies to ascertain the true effect of any individual factor. In our own experience, the most striking example is of an affected mother with severe scoliosis, wheelchair-bound, and a daughter with the same mutation who is of near normal height having had bisphosphonate treatment from infancy. However, an understanding that there can be considerable variability in OI phenotype with an identical genotype even within the same family [43] should always be a caveat to such anecdotes; ascribing “better” outcomes to pharmacological interventions without a placebo-controlled trial is unwise.

There is no clear evidence from controlled trials that bisphosphonate use alters the natural history of skull base deformity and related complications, such as reducing the risk of basilar invagination requiring surgery. However, longer duration of bisphosphonate use was associated with a reduced odds ratio of developing basilar impression or invagination in a retrospective cohort review [44].

Adverse effects that have been widely reported include the acute phase reaction, seen in almost all older children following the first administration of either pamidronate or zoledronic acid, comprising fever up to 38.5 °C, musculoskeletal aches and pains, headache, nausea, and, rarely, vomiting and a rash [45]. This can be ameliorated by concomitant administration of paracetamol in standard doses for 48 h. Symptomatic hypocalcaemia is uncommon, and usually avoided by encouraging regular consumption of calcium-rich food or drink, typically dairy products (e.g. milk), but should be watched for in any child who is vomiting. Hypophosphataemia has been reported and appears to be dose-related in those receiving zoledronic acid; clinical sequelae have not been particularly evident in terms of functional bone or muscle outcomes [46].

More seriously, acute deterioration in respiratory status has been seen in a number of infants following the first administration of pamidronate [47]; it has become routine in some centres to admit infants to a high dependency unit for their first treatment.

Observational cohort studies have suggested that fracture healing is not impaired by the use of pamidronate, but that osteotomy healing may be slowed after three years-worth of treatment [48]. However, a number of factors can impact on bone healing, and it is possible that some, such as the routine use of non-steroidal anti-inflammatory agents for pain relief (such as diclofenac) [49], may not have been accounted for in these studies. Callus remodelling might be expected to be slowed by bisphosphonate treatment [50], but there is no evidence that the incidence of non-union is increased in bisphosphonate-treated children.

Over-dosing with bisphosphonates can result in osteopetrosis-like features. Whyte and colleagues originally reported on a 12-year-old boy without OI who had received approximately four times the normal annual dose of pamidronate over the period of 2.75 years and developed club-like metaphyseal deformities that persisted into adulthood [51]. More recently the group reported on a cohort of children with severe OI from India who had received high doses (4 mg at short intervals) of zoledronic acid and developed metaphyseal widening and sclerosis [52]. An increased metaphyseal index has been reported in relation to bisphosphonate use more generally, but to what extent this alteration in modelling persists or is harmful in itself remains unclear [53].

Whilst risk of atypical femoral fractures play a role in determining duration of bisphosphonate treatment in the management of post-menopausal osteoporosis, this experience cannot and should not be extrapolated to the paediatric OI population in a simplistic manner. Certainly, fractures resembling atypical femoral fractures can occur in children with OI who have not had bisphosphonate treatment. A retrospective study of fractures resembling atypical femoral fractures in non-deformed femurs of children with OI showed that there was no association with prior bisphosphonate treatment [54]. Rather, there was an association with severity of OI. The same study found that OI type VI was overrepresented in the cases with “atypical femoral fractures”. This accords with the personal clinical experience of the authors who have seen such fractures in cases of OI type VI in the absence of bisphosphonate treatment. The literature on atypical femoral fractures in children with OI also includes a case of a child who had received several years of pamidronate treatment but who also had a mutation close to the C-propeptide cleavage site [55]. Such mutations may predispose to a high bone mass OI phenotype in which bones are excessively brittle (even compared to other types of OI), perhaps making them at higher risk of such fractures.

Importantly, back scattered electron imaging studies have shown no increase in the tissue mineralisation density distribution consequent on bisphosphonate use [56].

There have been no recorded cases of osteonecrosis of the jaw in any child with OI, despite the high doses of pamidronate (compared to adult practice) that have been used in many [57].

There is no clear evidence as yet that bisphosphonate treatment in childhood improves hearing outcomes in OI, although one study using systematic methods to assess hearing loss reported a lower incidence of hearing loss compared to historical reports from the era before bisphosphonates were widely used [58].

Although renal complications have been observed in some adults receiving high dose pamidronate, this has not been reported in children with OI.

The effect of bisphosphonates on reducing bone remodelling activity is well reported and evidenced both with circulating biomarkers and on bone histomorphometry [17, 18]. One consequential concern is that reducing bone remodelling could have an adverse impact on the skeletal response to physiotherapy and mechanical stimulation. A small observational study recently reported significant reductions in both bone formation and resorption measured by circulating biomarkers in response to whole body vibration following only six weeks of risedronate treatment [59]. Whilst these data point to a potential adverse effect of bisphosphonates on bone physiology, the results need confirming in other larger studies conducted over longer time periods and need to be considered alongside the totality of evidence for benefit and harm of these drugs in children with OI.

### Denosumab

The initial reports of the use of denosumab in children with OI came from Germany, where four patients with severe OI type VI due to mutations in *SERPINF1* were moved from neridronate to denosumab due to apparent ineffectiveness of the bisphosphonate in improving bone mass or reducing bone turnover [60]. Denosumab treatment was given 1 mg/kg s/c at 3 monthly intervals. The report detailed suppression and then rebound of bone resorption monitored by urinary deoxypyridinoline crosslink/creatinine ratio. Subsequent reports on the progress of these children were restricted by two of the four returning to their country of origin; the remaining two showed some increase in their LSaBMD Z-score and some improvement in the height of crush fractured vertebrae [61].

A further prospective study of denosumab 1 mg/kg s/c 12 weekly in 10 children with mutations in *COL1A1* or *COL1A2* was reported in 2016 and found significant increases in LSaBMD Z-score – + 0.96SDS at 48 weeks—but no change in vertebral morphometry or functional outcomes, although there was a strong trend towards an improved six minute walk test in those who were mobile. The expected changes in bone turnover markers and bone metabolism parameters were observed. There was one episode of hypocalcaemia, and no episodes of accelerated bone turnover post-treatment [62].

A large-scale open-label study (NCT02352753) of denosumab was sponsored by its manufacturer, and recruited 153 children aged 2–17 years. The results have been published on clinicaltrials.gov, but not formally reported as yet in a peer-reviewed journal.

The initial study regimen was 1 mg/kg s/c up to a maximum of 60 mg every 6 months for 3 years. Patients were then transitioned to the same dose every 3 months at any time up to month 36 at the discretion of the local Principal Investigator. A large number of subjects withdrew from the study (*n* = 34), 2 were lost to follow-up, and 2 were withdrawn by the sponsor. 60 subjects transitioned from 6 to 3 monthly dosing prior to month 12. Of these 60, 40 completed 12 months of q3 monthly dosing treatment.

Change in LSaBMD Z-score at month 12 in the q3 monthly group was + 1.009 SDS, and + 0.925 SDS at 6 months. Long bone or new vertebral fracture incidences for the last 12 months of the q6 monthly dosing regimen, or for month 12 of the q3 monthly dosing regimen were similar at 28.3% and 26.7% of participants. Improvements in vertebral fracture were seen in 28.3 and 30.4% in each group, respectively. The expected changes in bone turnover markers were observed. No changes in functional outcome or growth were seen.

The study was stopped in late September 2021 due to safety concerns after eight children in the q3 monthly dosing group developed severe hypercalcaemia. Others have reported hypercalcaemia and hypercalciuria during denosumab treatment of children with OI [63].

## Anabolic Pharmacological Interventions in Children with OI

### Anti-Sclerostin Antibodies

There are two on-going studies of setrusumab in children, one phase 3 comparator study in younger children aged two to less than seven years (NCT05768854) and one phase 2/3 study (NCT05125809) enrolling children and young adults aged 5–25 years. The phase 2 part of the latter study (comparing 20 mg and 40 mg/kg monthly infusions) is complete at the time of writing, with the phase 3 part still recruiting at a 2:1 ratio of setrusumab 20 mg/kg versus placebo. There are no peer-reviewed publications of setrusumab data for children. The phase 2 data have been presented in abstract form only. Adults with OI treated with the drug over a 21 week period in an open-label study showed an increase of 4.1% in LSaBMD.

An open-label ascending dose (up to 3 doses) study (NCT04545554) of the subcutaneously administered anti-sclerostin antibody romosozumab in children aged 5–18 years with OI has been undertaken but not yet reported. A large-scale study of romosozumab (NCT05972551) is now planned but not yet recruiting.

### Parathyroid Hormone

There have been no studies of parathyroid hormone in children with OI; there is a “black box” warning against the use of parathyroid hormone and parathyroid hormone-related peptide in children, based on the occurrence of osteosarcomas in growing rats receiving these interventions.

It is possible that parathyroid hormone might be used in those whose growth plates have fused but are still legally considered children, whilst noting that the legally defined end of childhood is not consistent across the world.

A randomised placebo-controlled trial of PTH for 18 months in 79 adults with OI of varying severity—51 mild, 26 moderate-severe—showed the expected initial increase in bone formation over the first 12 months with subsequent decline, accompanied by similar changes in bone resorption. LSaBMD increased by 6.1% in the PTH-treated group vs 2.8% in those receiving placebo; the difference in those with type I after adjusting for other factors was 5.1%, but not significant in those with more severe OI. The difference at the total hip was similar (5.0%) in those with type I OI. Those with more severe OI treated with PTH showed no difference at any timepoint to those receiving placebo [64].

The TOPAZ trial—Treatment of Osteogenesis Imperfecta with Parathyroid hormone and Zoledronic acid (NCT03735537) has recruited 350 patients across 29 sites in 5 countries. The study compares two years of teriparatide followed by zoledronic acid with standard care, with the primary outcome being number of participants with new clinical fracture confirmed by imaging (information provided by Professor Stuart Ralston). The study is powered to detect a 25% reduction in risk of fracture validated by imaging in the teriparatide-treated as opposed to standard care group. It is hoped that study results will be available in Q2/3 of 2025.

## Anti-Transforming Growth Factor Beta Approaches

### Anti-Transforming Growth Factor Beta Antibodies

There have been no reports of the use of any anti-transforming growth factor beta (TGFb) antibody treatment in children with OI; the report of the first use of fresolimumab in adults (NCT03064074) did include analysis of bone biopsies from four children that showed increased TGFb pathway signalling activity [65]. Other antibodies against TGFb are in early stage development; Sanofi-Aventis SAR439459 is in phase I/II with a single dose ascending study underway in adults with OI (https://www.sanofistudies.com/us/en/listing/295191/single-ascending-dose-study-of-sar439459-in-adults-with-osteogenesis-imperfecta-oi/).

### Losartan

A study to assess the effect of different doses of losartan in older adolescents and adults with OI is in preparation (ISRCTN13317811) as part of the REMEDi4ALL consortium (HORIZON-HLTH-2021-DISEASE-04-02 Proposal number: 101057442), with plans to recruit 30 patients across six sites in the UK and Italy. Enrolment is expected to begin in early 2024.

## Other Bone-Directed Treatments

### Fluoride

Some case reports of the use of fluoride in OI are present in the literature from 50 years ago [66, 67]; there have been no published larger-scale studies.

### Growth Hormone

Marini and colleagues reported on the use of recombinant growth hormone 0.1–0.2 IU/kg six times per week for at least 1 year in 26 children with moderate and severe OI aged 4.5–12 years in an open-label prospective study [68]. The authors divided the group into “responders” and “non-responders” depending on change in growth rate; “responders” showed an increase in growth rate of 50% or more compared with pre-treatment growth rate. Responders were reported to have a higher growth rate overall compared to non-responders (6.4 ± 2.0 vs. 4.0 ± 1.7 cm/yr), and lower fracture rate compared to the pre-treatment 12-month period (1.21 ± 0.7 vs. 2.0 ± 1.1 fractures/yr). The serum carboxy-terminal propeptide of type I collagen concentration increased more in the responders than the non-responders; there was no difference between groups in the circulating level of bone-specific alkaline phosphatase. LSaBMD measured using DXA showed a 5–7% increase in LSaBMD z-score in the responders and 3–5% in the non-responders. Bone biopsy data from 20 of the children showed an increase in cancellous bone volume (BV/TV) and trabecular number as well as in surface extent of mineralisation (MS/BS), and consequently bone formation rate (BFR/BS and dLS/BS). Of note, the statistical analysis section of the methods did not clarify whether dividing the study cohort into two groups was pre-specified in the data analysis plan.

Antoniazzi and colleagues in a smaller study of 14 children with mild OI undertook a controlled study of daily subcutaneous growth hormone 0.6 IU/kg/week in 7 children, with seven followed as controls [69]. They reported a significant increase in growth rate (6.04 ± 0.69 vs. 4.02 ± 0.31 cm/yr) during the 12-month period of treatment; height velocity was similar in the year following treatment between the groups. They also reported an 11.4% increase in calculated volumetric BMD of the lumbar spine based on anteroposterior and lateral DXA measurements; measurements 12 months post-treatment were not reported.

None of the growth hormone studies reported followed children to final height.

## Other Pharmacological Interventions

### Indomethacin

Indomethacin has been used in Type V OI to reduce hyperplastic callus formation following fracture and osteotomy. No randomised studies have been undertaken. The dose used is typically 2 mg/kg/d in divided doses, given with food, over a two to three week period. Longer periods of administration have been recorded in individual cases where callus formation has been massive. However, Cheung and colleagues reported there was no evidence in their observational series that indomethacin administration had a beneficial effect on hypertrophic callus formation [70]. Cover with a gastro-protective agent should always be considered alongside indomethacin, and particular caution should be exercised if a child is on an oral bisphosphonate.

### Pain Management

Some centres involve their pain management team as part of their overall multidisciplinary approach to OI; generally, the advice is the same as for any chronic pain syndrome, to start with simple analgesia such as paracetamol or ibuprofen, alone or in combination. Opiate use is usually restricted to acute fracture-related pain episodes and perioperative management. Some individuals state that they get relief of bone pain from the administration of bisphosphonates, although this has not been substantiated in placebo-controlled trials. Physiotherapy-based approaches to managing pain are essential given the high incidence of pain due to muscle and other soft tissue problems. Soft tissue problems may be the sole cause of back pain in some cases, and it is important to avoid a bias towards medical solutions in all situations.

### Tranexamic Acid

Tranexamic acid has been shown to reduce blood loss during operative procedures, and may shorten post-operative hospitalisation [71].

Post-operatively, avoidance of non-steroidal anti-inflammatory drugs may be of value in aiding healing of osteotomy sites.

## Cell-Based Therapies

One basic tenet underlying the use of cell-based therapy in OI is that full replacement of defective cells is not necessary, as studies in two individuals who were mosaic for different type I collagen mutations suggest that normal skeletal function can be maintained with between 40 and 75% of osteoblasts carrying the mutation [72]. Another is that the provision of mesenchymal stem cells (MSCs) will provide daughter osteoblasts that integrate into the overall skeletal population of metabolically active cells and are capable of producing normal type I collagen that is deposited in the skeleton.

Different groups have adopted different strategies in this area. The earliest reports of MSC administration came from the group of Horwitz; the group published initially on their experience of allogeneic bone marrow transplantation—either HLA identical or single mismatch sibling donor-derived—in three children [73] with severe OI who had undergone standard pre-transplantation ablative conditioning. In the only child in whom there was both high (> 99%) haematopoietic engraftment and from whose post-transplant bone biopsy (day 80) osteoblasts could be grown, only 2.0% of osteoblasts were from the donor. Significant increases in bone mineral content were reported, but it was unclear whether this was from increased bone formation or reduced resorption following marrow ablation. All the patients were subsequently given pamidronate.

These patients and three others were subsequently infused (twice each) with mononuclear cells with mesenchymal cell characteristics that had been transduced with either the G1PLII or LNc8 retroviral vectors after their first passage in culture [74]. These cells were taken from the original bone marrow transplant donors, cultured under standard conditions and identified primarily on their adherence to plastic after three days. Aliquots of the cells showed mineralisation nodules on Alizarin red staining and exposure to osteogenic media. No data were provided on specific markers such as osteocalcin. No ablation was undertaken, but hydrocortisone, acetaminophen (paracetamol), and diphenhydramine were given pre-infusion. One patient had an infusion-associated reaction with urticaria which resolved with further hydrocortisone and diphenhydramine.

Engraftment, based on detection of the retroviral vector markers, was shown in 5 of 6 patients, but never exceeded 1% in any of skin, bone, or bone stromal tissue. 5 of 6 patients showed significant increases in growth velocity post-infusion, but no change in bone mass by DXA.

Subsequently, there have been two reported cases in which human foetal stem cells were administered antenatally to infants known to carry mutations likely to cause moderate to severe OI, with additional postnatal infusion of MSCs from the same donors [75]. The authors reported good safety profiles. Efficacy is difficult to assess in such open-label studies; the first patient suffered multiple long bone fractures, as well as 11 vertebral compression fractures and scoliosis from age 6 years. Reinfusion of MSCs at age 7.75 years was associated with an apparent cessation of fractures for the following two years along with improved mobility and motor function generally, but other interventions such as intramedullary rodding were performed, and the patient also received bisphosphonates. The second patient received a postnatal reinfusion of MSCs at age 19 months; no postnatal fractures were reported, although there was clear evidence of antenatal fractures. This patient also received bisphosphonates. The authors reported on another infant with the same mutation as the first who received MSCs—that child died from pneumonia at age 19 weeks having been started on zoledronic acid at age day 6 of life.

In the TERCELOI study (NCT02172885), adult-derived MSCs were infused five times over a period of 2.5 years with further follow-up over two more years in two patients aged six and eight years [76]. The MSCs were prepared from bone marrow aspirates from HLA-haploidentical siblings by separating and purifying the mononuclear cell fraction. No infusion-related reactions were reported. Both patients had had fractures in the year prior to treatment and both had fewer fractures during the period of the infusions. Both showed increases in LSaBMD although to a different degree; additionally, the reported increases in aBMD were similar to those that might be expected over the same time period in a child receiving bisphosphonates. The study reported improvement of trabecular bone BV/TV at one year post-infusion in the distal femur of both patients based on the use of QUIBIM software, although this was not maintained at two years. Detailed analysis of the proteins expressed and circulating in patients’ sera after successive MSC infusions showed significant changes in multiple areas; the authors postulated that MSC infusion resulted in a pro-osteogenic paracrine response.

The BoostB4 study (NCT03706482; https://www.boostb4.eu/) is “An exploratory, open label, multiple dose, multicentre phase I/II trial evaluating safety and efficacy of postnatal or prenatal and postnatal administration of allogeneic expanded foetal mesenchymal stem cells for the treatment of severe Osteogenesis Imperfecta compared with a combination of historical and untreated prospective controls.” The study opened at the end of 2019, so recruitment has been impacted by the COVID pandemic. Nevertheless, recruitment to the postnatal part of the study closed in February 2022.

It is very difficult currently to determine the efficacy of cell-based interventions since the studies are necessarily open label, observational, have not omitted standard of care, have a very variable baseline, and there has been significant uncertainty as to the degree of engraftment; the results of the BoostB4 study are nevertheless keenly awaited.

## Other Approaches Still at Preclinical Stage

Combination anti-activin and myostatin antibodies alone or in combination have been assessed in preclinical studies [77, 78]. Use of a prostaglandin E2 receptor 4 (EP4) agonist covalently linked to alendronate has been studied in the jrt mouse model [79]. A multitude of other approaches, including autophagy enhancement [80], a lipidomic (oleoyl serine derivative), and a microRNA targeting approach have come up on recent horizon scans; gene therapy for the majority of cases where *COL1A1* and *COL1A2* mutations are causative remains a distant prospect with the currently available technologies.

## Summary

Having had over three decades in which the options available to treat OI have been limited to bisphosphonates with questionable efficacy, multiple new approaches are now under development. The application of such approaches in the real world—in patients who have previously been exposed to bisphosphonates, in infants with fractures occurring in utero or around the time of delivery, and in patients with OI due to non-collagen mutations—will require continuing multicentre, multinational collaborative efforts. We hope that our patients and their families will fully realise the potential benefits that these new approaches may bring over the coming years.
